# Repair of bone defects using adipose-derived stem cells combined with alpha-tricalcium phosphate and gelatin sponge scaffolds in a rat model

**DOI:** 10.1590/1678-77572016-0094

**Published:** 2017

**Authors:** Adriana CORSETTI, Claudia BAHUSCHEWSKYJ, Deise PONZONI, Renan LANGIE, Luis Alberto dos SANTOS, Melissa CAMASSOLA, Nance Beyer NARDI, Edela PURICELLI

**Affiliations:** 1 Universidade Federal do Rio Grande do Sul, Faculdade de Odontologia, Porto Alegre, RS, Brasil.; 2 Irmandade da Santa Casa de Misericórdia de Porto Alegre, Centro de Odontologia, Cirurgia e Reabilitação Bucomaxilofacial, Porto Alegre, RS, Brasil,; Universidade Luterana do Brasil, Laboratório de Células-Tronco e Engenharia de Tecidos, Canoas, RS, Brasil.; 3 Universidade Federal do Rio Grande do Sul, Departamento de Engenharia de Materiais, Porto Alegre, RS, Brasil.; 4 Universidade Luterana do Brasil, Laboratório de Células-Tronco e Engenharia de Tecidos, Canoas, RS, Brasil.; 5 Irmandade da Santa Casa de Misericórdia de Porto Alegre, Centro de Odontologia, Cirurgia e Reabilitação Oral e Maxilofacial, Porto Alegre, RS, Brasil

**Keywords:** Bone regeneration, Calcium cement, Adipose-derived stem cells, Rat model

## Abstract

**Objectives:**

This study aimed to evaluate the potential of adipose-derived stem cells (ASCs) combined with a modified α-tricalcium phosphate (α-TCP) or gelatin sponge (GS) scaffolds for bone healing in a rat model.

**Material and Methods:**

Bone defects were surgically created in the femur of adult SHR rats and filled with the scaffolds, empty or combined with ASCs. The results were analyzed by histology and histomorphometry on days seven, 14, 30, and 60.

**Results:**

Significantly increased bone repair was observed on days seven and 60 in animals treated with α-TCP/ASCs, and on day 14 in the group treated with GS/ASCs, when compared with the groups treated with the biomaterials alone. Intense fibroplasia was observed in the group treated with GS alone, on days 14 and 30.

**Conclusions:**

Our results showed that the use of ASCs combined with α-TCP or GS scaffolds resulted in increased bone repair. The higher efficacy of the α-TCP scaffold suggests osteoconductive property that results in a biological support to the cells, whereas the GS scaffold functions just as a carrier. These results confirm the potential of ASCs in accelerating bone repair in *in vivo* experimental rat models. These results suggest a new alternative for treating bone defects.

## Introduction

The management of lost bone tissue due to congenital abnormalities, trauma, or cancer treatment poses a challenge to oral and maxillofacial surgeons. The highly vascularized nature of the bone tissue results in a great capacity to heal and remodel without scarring^8^. Nevertheless, bone loss represents a major clinical problem in reconstructive head and neck surgery, and autologous grafting is still the therapeutic gold standard in reconstructive surgery. This concept, however, has serious limitations related to the limited amount of tissue that can be harvested, increased risk of infection, or recurrent pain. Alternative therapeutic approaches have proposed osteoconduction, guided bone regeneration, osteodistraction, and osteoinduction^4^.

Osteoconductive scaffolds create an environment permissive for the proliferation of bone cells, which fills the bone defect^10^. Calcium phosphate cements (CPCs) have a composition similar to the mineral phase of native bone, and have been used as bone substitutes in the last decades^15^. Hydroxiapatite and tricalcium phosphates are among the most used of these cements, and they have proved their value in several clinical applications^6^. However, CPCs have some limitations, related to poor mechanical properties and mainly to lack of osteoinductive and angiogenic activities. The first limitation can be dealt with by further modification of the biomaterial. To compensate for the lack of osteoinduction and angiogenesis, CPCs have been preloaded with cells.

The combination of adult stem cells with biomaterials has introduced new perspectives on the optimization of tissue repair protocols. Differentiated cells or stem cells may be used, and mesenchymal stem cells are among the most extensively studied biological elements in tissue engineering^7^. The plasticity and ease of collecting and *ex vivo* culturing of adipose-derived stem cells (ASCs) open wide possibilities of use in regenerative therapy^2^. This study aimed to evaluate the process of bone repair in a rat femoral defect model, using modified α-tricalcium phosphate (α-TCP) scaffolds and a commercially available biomaterial (absorbable gelatin sponge, GS) associated with allogeneic ASCs. Results were evaluated by histological and histomorphometric assessments of bone repair.

## Material and methods

### Animals and ethics

Adult (5 months old) male syngeneic SHR rats, weighing an average of 300 g, were housed and maintained under standard conditions, and treated in accordance with the guidelines for the use of animals in research projects, Normative Resolution 04/97 of the Research and Ethics in Health Committee/GPPG/HCPA. The study was approved by the Research Ethics Committee of Hospital de Clínicas de Porto Alegre (no. 110159).

### Isolation and characterization of adipose-derived stem cells

Inguinal adipose tissue was collected from three rats and processed individually as previously described^1^. The tissue was minced in phosphate buffered saline (PBS) and digested with 1 mg/mL collagenase type I solution (Sigma Chemical Co, St Louis, MO) for 30 min at 37°C, under gentle agitation. The enzyme was inactivated with Dulbecco’s modified Eagle’s Medium (DMEM) (Sigma) supplemented with 10% fetal calf serum (FCS, Cultilab, SP, Brazil) and centrifuged at 400x g for 10 min. The vascular stromal fraction was washed with Hank’s balanced salt solution (HBSS) (Sigma) by centrifugation at 350x g for 5 min. The cell pellet was resuspended in DMEM with 10 mM HEPES free acid (Sigma) supplemented with 10% FCS and cultured at 37°C in an atmosphere of 5% CO_2_. Three days later, the non-adherent cells were removed, and the culture was maintained and expanded every 3 or 4 days after trypsinization (0.25% trypsin and 0.01% EDTA in HBSS). Cells between passages 4 and 7 were used for all experiments, and at least 3 cultures were analyzed.

ASCs were analyzed for morphology, immunophenotype, and proliferation and differentiation potential. All experiments were reproduced three times. Photomicrographs were taken with a digital camera (AxioCam MRc, Carl Zeiss, Oberkochen, Germany), using AxioVision 3.1 software (Carl Zeiss). The immunophenotype was determined by flow cytometry. The cells were trypsinized, washed, and incubated for 30 min at 4°C with fluorescein isothiocyanate (FITC)-conjugated antibodies specific for rat CD29, CD44 (Becton Dickinson, San Jose, CA), CD11b and CD45 (Caltag, Burlingame, CA). A FACSCalibur flow cytometer equipped with 488 nm argon laser (Becton Dickinson, San Diego, CA) was used with the CellQuest software. At least 10,000 events were collected.

The proliferation rate of the cultures was assessed by counting the number of cells recovered in each passage, as well as the time elapsed. These data were used to determine the population doubling time, with the aid of an online calculator (http://www.doubling-time.com/compute.php). The results are expressed as the number of cells over the days of cultivation.

Differentiation was induced by incubation of cells with specific culture medium^1^. All reagents were from Sigma, unless specifically indicated. For osteogenic differentiation, cells were cultured for up to 8 weeks in medium complemented with 10^8^ M dexamethasone, 5 μg/mL ascorbic acid 2-phosphate, and 10 mM β-glycerophosphate. Calcium deposition was revealed by staining for 5 min with Alizarin Red S stain at pH 4.1. To induce adipogenic differentiation, cells were cultured for up to 8 weeks in medium complemented with 10^8^ M dexamethasone, 2.5 μg/mL insulin, 100 μM indomethacin, and 3.5 μM rosiglitazone. Adipocytes were revealed by staining with Oil Red O.

### Biomaterials

The α-TCP scaffolds were synthesized using calcium carbonate (Dinamica, SP, Brazil) and calcium pyrophosphate, obtained from calcination of calcium phosphate dihydrate (Dyne, SP, Brazil) at 550°C for 5 h. Initial dry homogenization was made in alumina ball mill for one hour. The mixture of powders were calcined in a furnace at 1300°C for 5 h, followed by quenching in air.

**Figure 1:**
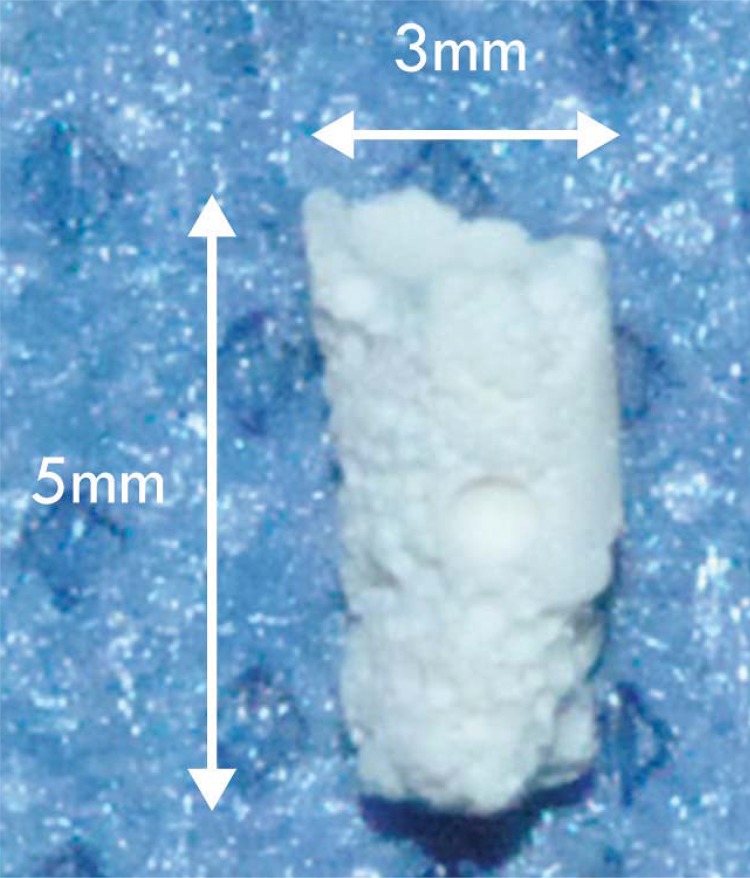
α-TCP scaffold dimensions (3 mm diameter and 5 mm length)

After cooling, α-TCP was manually disaggregated using porcelain mortar and the powder was sieved in #60ASTM mesh (250 μm). The sieved powder was wet milled using absolute ethyl alcohol (Vetec, SP, Brazil) in alumina ball mill for 2h. The resulting fine powder (average particle diameter 9 μm) was dried in a stainless steel vessel for 72 h at 70°C to promote the evaporation of alcohol. After milling, the α-TCP is called cement, as it reacts with water and allows hardening. The liquid fraction of the cement was distilled and deionized in water with cement setting accelerator (2.5% Na_2_HPO_4_ – Synth, SP, Brazil) and foaming agent (0.5% sodium dodecyl sulfate, Dinamica). The powder and liquid were hand-mixed to produce a foaming cement using a liquid/powder relation of about 0.5 mL/g. After hardening, the foaming cement was washed 5 times to remove the excess of foaming agent. The apparent porosity of obtained cement was 61%, with pores ranging from about 100 to 400 μm. Scaffolds used in all experiments were 3 mm diameter and 5 mm length (Figure 1).

A commercially available re-absorbable gelatin sponge (GS, Cutanplast Standard, SP, Brazil) was also used in the experiments. Both types of scaffolds were analyzed by scanning electron microscopy (SEM), using a microscope Hitachi, model TM3000.

### Combination of ASCs to biomaterials

ASCs were applied to the scaffolds by static seeding. Dry α-TCP cylinders (3 mm widex5 mm deep) or a corresponding volume of gelatin sponge were individually placed in wells of a 24-well culture plate. ASCs were suspended in DMEM at 5x10^6^ cells/mL, and 50 μL were placed on each scaffold. Adherence of ASCs to α-TCP scaffolds was determined by incubation for 2 h at 37°C, with addition of 10 μL DMEM at every 30 min, after which 200 μL of DMEM were added to each well and the scaffolds were transferred to new wells with DMEM complemented with 10% FCS. The remaining, non-adhered cells were stained with Giemsa and counted, to determine the number of cells adhered to each scaffold. For analysis of cell proliferation, the scaffolds were maintained for 3 d in 5% CO_2_ at 37°C, and proliferation was assessed using the MTT test^9^. Optical density was read in a spectrophotometer at a wavelength of 540 nm. The same number of cells combined with the scaffolds was cultivated in conventional conditions for 3 d and analyzed with the MTT test.

### Surgical procedure and treatment

**Figure 2:**
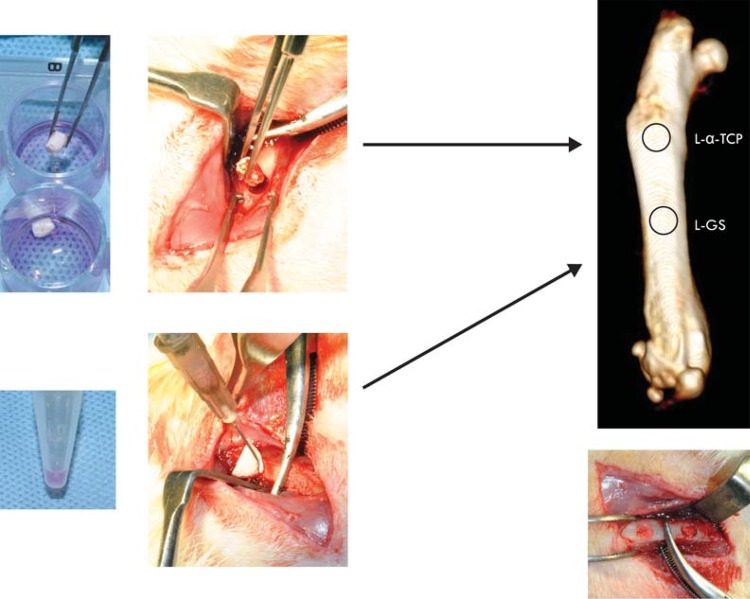
Transoperative procedures of test groups - treatment of the bone defect with ASC-loaded α-TCP scaffolds (L-α-TCP) and ASC-bone loaded gel sponge (L-GS)

Surgical procedures were performed under general anesthesia using intraperitoneal injections of 10 mg/kg body weight xylazine (Bayer, Newhaven, CT) and 50 mg/kg body weight ketamine (Parke-Davis, Ann Arbor, MI). As Figure 2 shows, two bone defects measuring 3.1 mm diameter and 3.5 mm depth were surgically created in the right femoral diaphysis, using a motorized trephine. In 16 rats, the two defects were totally filled with empty α-TCP or GS scaffolds (E-α-TCP and E-GS, respectively), and in another 16 animals, with ASC-loaded α-TCP or GS scaffolds (L-α-TCP and L-GS, respectively) prepared as described above. The animals were observed daily, according to usual veterinary post-operative care.

On days seven, 14, 30, and 60 after the surgical procedure, groups of eight rats (four with empty scaffolds and four with ASC-loaded scaffolds) were sacrificed. The right femurs were dissected and removed for analysis.

### Histology and histomorphometry

Implanted bone areas were collected, decalcified with 5% nitric acid for 5 d, dehydrated with graded ethanol and processed for histological analysis. Sections perpendicular to the implant and surrounding area were stained with hematoxylin and eosin and analyzed with a microscope (Olympus Optical Co, Miami, FL) connected to an image analyzer (Olympus, Qcolor 5, Coolet, RTV), using the Qcapture software (University of Texas). The size of the remaining bone defect was measured in pixels with the distance tool of the ImageTool program (Version 2.81, Quantitative Imaging Corporation), and a zero score was assigned to fully repaired bone defects. Repaired defects were categorized and shown as absolute values, and non-repaired defects were statistically analyzed as described below.

The amount of newly formed bone tissue was measured in percentage by drawing a line from the outer surface of a cortical layer, always with the same length. Other boundary lines were determined at right angles to this one, forming a rectangle. The smallest and largest sides of the rectangle measured 800 and 1200 pixels respectively. These measures were determined to keep a standardization and for being the largest area of new bone formation observed in the literature. The area of newly formed bone tissue was selected, excluding intertrabecular and vascular spaces as well as the remaining cortical layer, generating pixel values that were converted to a percentage value

Two blinded researchers conducted histomorphometric analyses, previously calibrated as shown by the Student’s t-test for paired samples and by the Bland-Altman plot.

### Statistical analyses

Data are expressed as mean and standard deviation. Differences among the groups were compared with the Student’s t-test for independent samples, using the SPSS for Windows version 19.0. A p value <0.05 was considered statistically significant.

## Results

### Cultivation and characterization of rat adipose-derived stem cells

**Figure 3:**
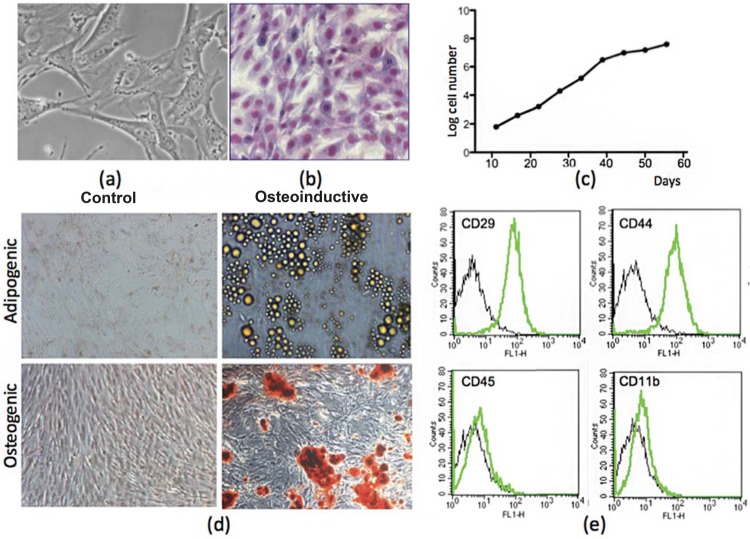
Morphology, proliferation, immunophenotype, and differentiation potential of rat adipose-derived stem cells (ASCs). Rat ASCs display the characteristic fibroblast morphology (a, in phase contrast; b, after Giemsa staining), and proliferation capacity (c) of mesenchymal stem cells. ASC cultures were able to differentiate into adipogenic and osteogenic lineages, as shown by staining with Oil Red O and Alizarin Red S, respectively (d), and were negative for CD11b and CD45 and positive for CD29 and CD44 (e). Undifferentiated (control) ASCs are not marked with these stains. Original magnification x200

As Figure 3 shows, cultivated rat ASCs show the characteristic fibroblast morphology and surface phenotype of mesenchymal-type stem cells. The cells expanded rapidly for a period of 40 d, when the expansion rate showed a decrease (Figure 3c). As expected, cultures differentiated into adipogenic and osteogenic lineages (Figure 3d).

### Production of α-TCP scaffolds and analysis of cell adherence and proliferation

**Figure 4:**
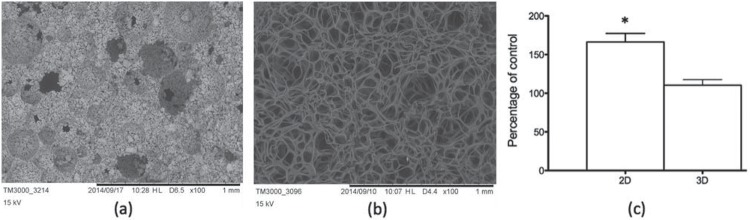
Scanning electron microscopy analysis shows the porous structure of α-tricalcium phosphate scaffolds (a) and the fibrous network structure of the gelatin sponge (b). After three days in culture, ASCs combined with α-TCP (3D) proliferate significantly less than cells plated in conventional conditions (2D), as shown by MTT results compared with the initial cell number (control). * p<0.05

The α-TCP scaffolds produced were analyzed by scanning electron microscopy (Figure 4a), which revealed adequate porous structure, with a pore size between 100 and 400 μm. SEM of samples of the gelatin sponge showed a fibrous network structure (Figure 4b). Rat ASCs had >90% adherence to α-TCP (not shown), and the proliferation rate of cells combined with the scaffolds was significantly lower than that of cells cultivated in conventional conditions (Figure 4c).

### Histological analyses

**Figure 5:**
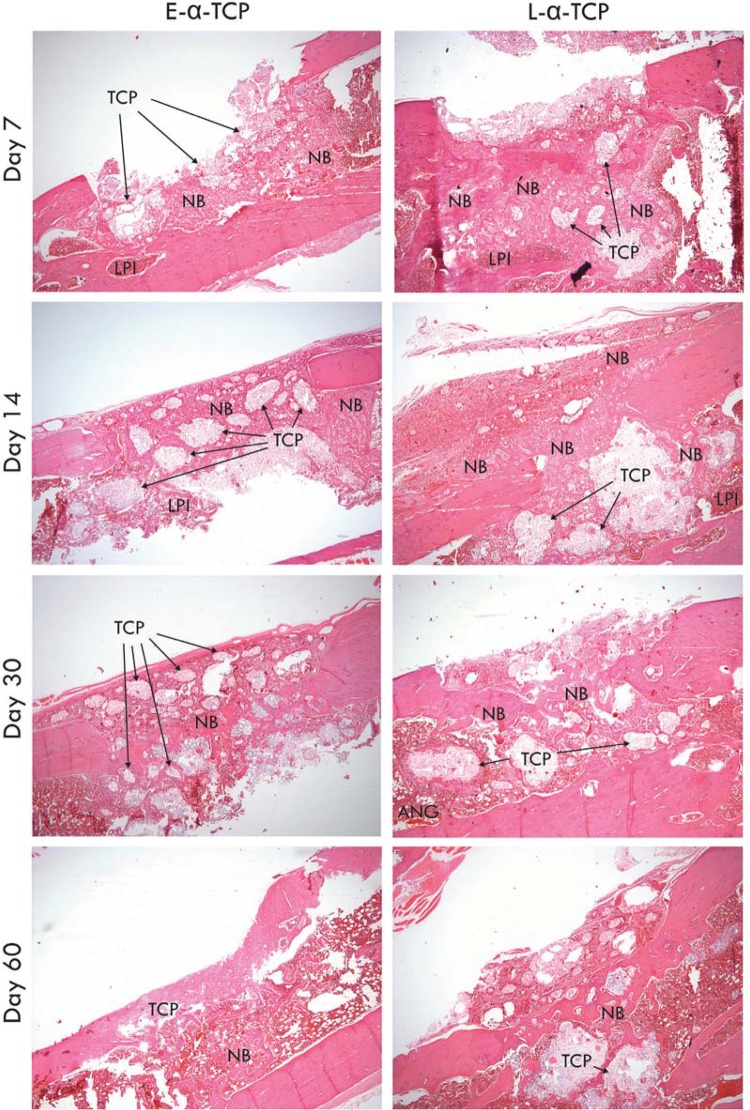
Treatment of the bone defect with ASC-loaded α-TCP scaffolds (L-α-TCP) results in faster repair as compared with defects treated with scaffolds alone (E-α-TCP). ANG, angiogenic region; LPI, lymphoplasmacytic infiltrate; NB, neoformed bone tissue; TCP, α-TCP. Magnification: x40

All histological evaluations showed a marked regular interruption of the ostectomized cortical bone. As presented in Figure 5, the treatment of bone defects with α-TCP scaffolds induced the formation of increasing amounts of new bone, particularly when the scaffolds were loaded with ASCs. Multinucleated giant cells were observed around the borders of the scaffolds, particularly on day 14. A lymphoplasmacytic infiltrate could be observed in some of the samples. Whereas in defects treated with E-α-TCP the new bone tissue was sparser and disorganized, the use of L-α-TCP resulted in new bone tissue that surrounded and progressively replaced the scaffolds, organizing and closing the surgical defect, with more frequent angiogenic regions.

**Figure 6:**
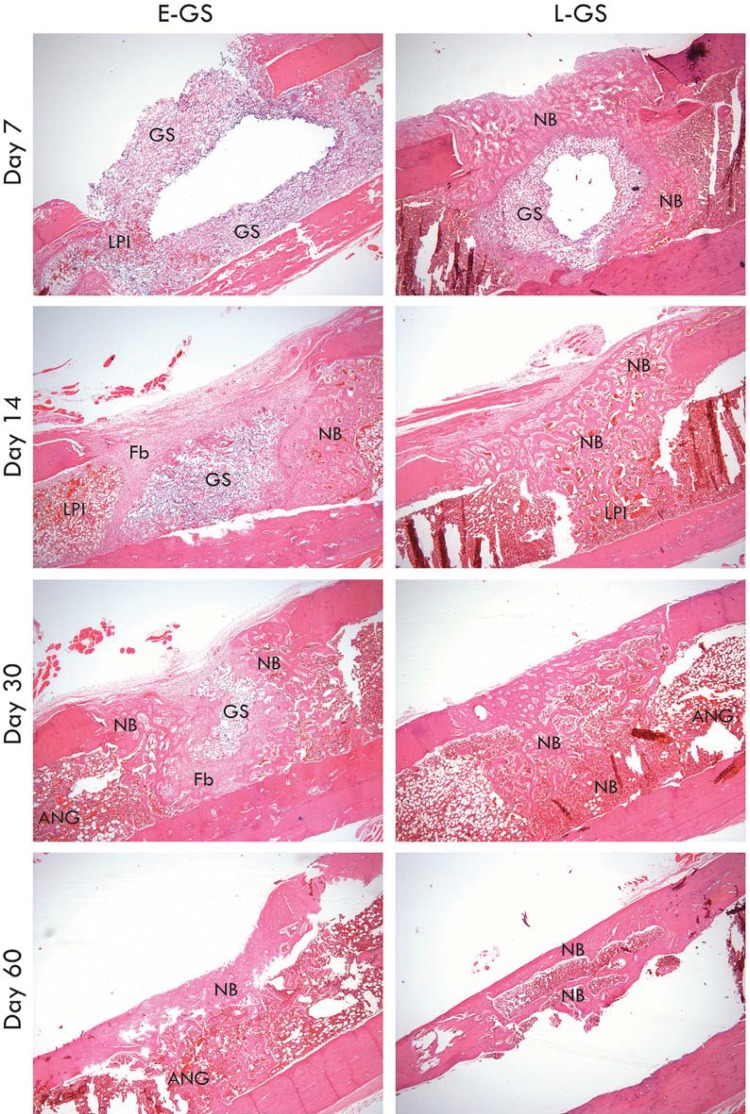
Extensive bone formation is observed in bone defects treated with ASC-loaded gel sponge (L-GS). Treatment with gel sponge alone (E-GS) also induced neoformation, but intense fibrosis is also observed. ANG, angiogenic region; Fb, fibrotic region; LPI, lymphoplasmacytic infiltrate; NB, neoformed bone tissue. Magnification: x40

Figure 6 shows the sequence of events observed after treatment of the bone defects with empty or ASC-loaded GS scaffolds. In defects treated with E-GS, the scaffold was observed up to 30 d; new bone tissue was first seen on day 14 and increased until most of the defect was repaired on day 60. However, intense fibrosis was also observed in these samples, particularly on days 14 and 30. The presence of ASCs induced extensive bone formation, observed on day seven and progressing until the bone defect was fully repaired. On day seven, L-GS scaffolds were already surrounded by massive trabecular bone with an inflammatory infiltrate containing predominantly polymorphonuclear cells. In these samples, the scaffolds were not visible after day 14.

### Histomorphometric analyses

Histomorphometric analyses evaluated the size of the bone defects on days seven and 60 after treatment. As presented in Table 1, the presence of adipose-derived stem cells resulted in a significant decrease of the mean size of bone defects treated with α-TCP scaffolds on day seven (p=0.016) or day 60 (p=0.042), as compared with defects treated with the scaffolds alone. On day 60, two of the four defects treated with ASC-loaded α-TCP scaffolds were completely repaired.

**Table 1:** Size of bone defect in pixels (size) and number of completely repaired bone defects (n rep) after treatment with α-tricalcium phosphate scaffolds (α-TCP) or gel sponge (GS) alone (empty) or loaded with ASCs (loaded)

	Day 7			Day 60		
	Empty	Loaded	p	Empty	Loaded	p
α-TCP size	1,537±218	702±454	0.016	492±19	288±58	0.042
n rep	0	0		0	2	
GS size	1,658±180	1,089±508	0.087	1,489±227	-	-
n rep	0	1		2	4	

For bone defects treated with the gel sponge, the presence of ASCs also reduced the mean size of the defect on day seven, but the difference was not significant. One of the defects treated with L-GS was already completely repaired on day seven. On day 60, two defects treated with E-GS, and all four defects treated with L-GS, were completely repaired.

The histomorphometric analysis showed a greater percentage of newly formed bone in the test cavities on days 7 and 60, but the differences were no statistically significant (Table 2).

**Table 2:** Size of bone defect in percentage (%) after treatment with α-tricalcium phosphate (α-TCP) or gel sponge (GS) scaffolds empty or loaded with ASCs, on days 7 and 60 after the procedure

	Day 7			Day 60		
	Empty	Loaded	p	Empty	Loaded	p
α-TCP	10.1±9.8	29.2±17.9	0.127	40.2±9.7	42.6±8.4	0.772
GS	7.3±2.5	19.2±19.2	0.303	36.4±11.8	36.8±6.5	0.959

## Discussion

This work investigated the efficacy of adipose-derived stem cells combined with two types of biomaterials, alpha-tricalcium phosphate and gelatin sponge, in the repair of bone defects in a rat model. The cells used in this study were easily isolated from the adipose tissue and expanded in culture, showing the characteristic fibroblast morphology and immunophenotype of mesenchymal-type stromal cells. They were also capable to differentiate into adipocytes and osteoblasts. Due to their self-renewal capacity and plasticity, ASCs represent an important component for regenerative medicine and tissue engineering^7^.

Although no consensus was reached about the ideal animal model to be used for the investigation of bone regeneration^5^, the experimental protocol used in this study, established by Puricelli, et al.^10^ (2010), has been very adequate for this type of study. The rats were all male, aged five months and weighing an average of 304 g, and the dimension of the surgical cavities were proportional to the length and thickness of the rat femur, measuring 3.1 mm in diameter and 3.5 mm deep. The ostectomized cortical structure showed marked regularity in all groups and experimental periods investigated. In addition, test and control cavities were distributed in the femur of different animals, preventing a possible effect of migrating stem cells in cavities that did not receive the cells.

Our results showed that the use of ASCs combined with α-TCP of GS scaffolds resulted in an acceleration of bone repair. This effect was mainly seen at seven and 60 days in the group treated with L-α-TCP and on day 14 when L-GS was used, resulting in greater bone neoformation and faster repair of the bone defect. Intense fibroplasia was observed in defects treated with gelatin sponge scaffolds alone, particularly on days 14 and 30. The higher efficacy of α-TCP scaffolds suggest an osteoconductive property that results in a biological support to the cells^10^, whereas the GS scaffold functions just as a carrier.

The gelatin sponge used in this study is a chemically cross-linked gelatin of high pore density^3^, and has already been successfully used alone or in combination with mesenchymal stem cells for bone tissue engineering. However, in those studies, the biomaterial was used just as a support for tissue implants^11^ or was combined with cells transduced with bone morphogenic protein-2^13^.

Although the osteoconductive properties of β-TCP scaffolds are well known, with an understanding of the signaling pathways through which they induce the differentiation of stem cells into osteoblastic cells^12^, less is known about α-TCP. In an *in vitro* study, Wójtowicz, et al. (2014)^14^ investigated the capacity of three types of CPCs to support the growth of human osteoblasts. The results showed that, although displaying cell-supporting properties, α-TCP scaffolds were less efficient than carbonate hydroxyapatite and biphasic calcium phosphate in inducing cell viability and spreading or osteogenic capacity of the osteoblasts. In this study, the results showed that ASCs have high adhesion to the cement scaffold. The smaller proliferation rate of the cells combined with α-TCP than in conventional culture conditions possibly indicate increased osteogenic differentiation, as shown by the accelerated bone repair when this combination was used. The biocompatibility of α-TCP, already observed by Puricelli, et al.^10^ (2010), resulted in absence of foreign body reaction in the treated tissues and increased reabsorption of the biomaterial, observed as early as seven days after treatment, with new bone formation at the edges of the implants.

For this study, modifications took place in the composition of the cement with the addition of surfactant sodium lauryl sulfate, resulting in the incorporation of pores in its structure and allowing the growth of bone tissue into the material. The block was porous and fragmented in histological sections. This may suggest that a larger area of contact with the bone tissue accelerates the process of repair, which could explain the accelerated process of bone repair in this study when compared with the results by Puricelli, et al.^10^ (2010), in which control cavities remained open 60 d after treatment.

Histomorphometric results are generally presented as the percentage of neoformed tissue in relation to the total defect, but, in this study, the size of the cavities were also measured, on days 7 and 60. The percentage of newly formed bone tissue showed no significant difference in treated and control groups, but the linear analysis of cavity occlusion showed significant results, with increased repair of the cavities when α-TCP was combined with ASCs.

These results suggest and confirm the potential of ASCs in accelerating bone repair in experimental rat models. Several authors agree that the association of a ceramic biomaterial to stem cells, from various origins, seems to favor and accelerate the process of bone repair, as observed in this study in experimental times seven and 60 d.

## Conclusions

This study showed through descriptive histological analyses that the combination of adipose-derived stem cells to two different types of biomaterials resulted in acceleration of bone repair. Calcium cement scaffolds were more efficient than gelatin sponge scaffolds, suggesting an osteoinductive function in addition to a cell-carrier function. These results should be further explored in larger animal models so that bone tissue engineering may soon become part of the therapeutic arsenal to treat an increasing number of patients in the world.
